# The segmentation of bones in pelvic CT images based on extraction of key frames

**DOI:** 10.1186/s12880-018-0260-x

**Published:** 2018-05-22

**Authors:** Hui Yu, Haijun Wang, Yao Shi, Ke Xu, Xuyao Yu, Yuzhen Cao

**Affiliations:** 1 Department of Biomedical Engineering, Tianjin University, Tianjin, China; 2 Department of Orthopedic, Tianjin Medical University General Hospital, Tianjin, China; 3 Department of Radiation Oncology, Tianjin Medical University Cancer Institute & Hospital, Tianjin, China

**Keywords:** CT segmentation, Pelvis, Key frame extraction, Skeleton, Marker-based watershed algorithm

## Abstract

**Background:**

Bone segmentation is important in computed tomography (CT) imaging of the pelvis, which assists physicians in the early diagnosis of pelvic injury, in planning operations, and in evaluating the effects of surgical treatment. This study developed a new algorithm for the accurate, fast, and efficient segmentation of the pelvis.

**Methods:**

The proposed method consists of two main parts: the extraction of key frames and the segmentation of pelvic CT images. Key frames were extracted based on pixel difference, mutual information and normalized correlation coefficient. In the pelvis segmentation phase, skeleton extraction from CT images and a marker-based watershed algorithm were combined to segment the pelvis. To meet the requirements of clinical application, physician’s judgment is needed. Therefore the proposed methodology is semi-automated.

**Results:**

In this paper, 5 sets of CT data were used to test the overlapping area, and 15 CT images were used to determine the average deviation distance. The average overlapping area of the 5 sets was greater than 94%, and the minimum average deviation distance was approximately 0.58 pixels. In addition, the key frame extraction efficiency and the running time of the proposed method were evaluated on 20 sets of CT data. For each set, approximately 13% of the images were selected as key frames, and the average processing time was approximately 2 min (the time for manual marking was not included).

**Conclusions:**

The proposed method is able to achieve accurate, fast, and efficient segmentation of pelvic CT image sequences. Segmentation results not only provide an important reference for early diagnosis and decisions regarding surgical procedures, they also offer more accurate data for medical image registration, recognition and 3D reconstruction.

## Background

Recently, traffic accidents, falls and other serious high-energy trauma have often led to pelvic fractures. Researches show that the incidence of heavy-vehicle injury has increased over the years [1] and patients with pelvic fractures who present in shock have a mortality of 30–50% [2]. Pelvic fracture is the third most common cause of death in traffic trauma [3]. Rapid and accurate diagnosis and treatment are not only important to reduce the mortality caused by pelvic fractures, but also helpful for the functional reconstruction and correction of deformities of the pelvis. Medical imaging and processing are crucial in the process of diagnosis and treatment. The computed tomography (CT) images and 3D reconstruction by CT are commonly used to display the anatomical structure of pelvis and characteristics of the lesions [4]. Analysis based on CT images is important for the description of pelvic anatomy, the planning of surgical procedures and the evaluation of the post-operative effects [5]. And the most important step of analysis is bone segmentation, which is crucial to quantitatively evaluate the degree of fracture, detect the location of bleeding, and judge the condition of injury [6]. Therefore, the accuracy of segmentation will affect the doctor’s judgment of the disease, the selection of the best surgical approach, etc.

At present, the pelvic region is manually marked for surgical procedures in the clinic. This process is time-consuming and error prone [7]. Therefore, it is necessary to propose a fast, accurate, and efficient method for pelvic segmentation.

Image segmentation is a hot topic in medical image processing. Recently, various segmentation methods have been proposed [8], some of which are based on thresholding [9–12]. The central idea of threshold segmentation is to transform the segmentation problem into pixel classification problem. Considering the fact that bone mineral density is heterogeneous, the connection between the femoral head and acetabulum is narrow, and a weak edge can be caused by disease, it is difficult to select a universal threshold. Other methods are based on the region growing technique, in which pixels with similar properties are set up as a region [13–15]. However, this method requires a long time and a large amount of space. In recent years, classification and clustering have also been used for medical image segmentation, and the relevant research focuses on the improvement of robustness [16–18]. Methods based on deformable models [19–25] and active shape models [26–30] have become a hot topic. For example, Calder et al. [20]proposed a segmentation method based on level set. Truc et al. [21] included density distance enhancement in a C-V model, and Martinez et al. [22] applied multi-scale edge detection to adjust a geometric model. Wu et al. [26] combined the active shape model with template matching, and Li et al. [27] combined the active shape model with a clustering model. However, most segmentation algorithms put more attention to the characteristics of a single CT image, so that the characteristics of CT sequences are usually ignored. Although there are many existing algorithms for medical image segmentation, an effective and accurate algorithm for the segmentation of pelvic CT image sequences has not been proposed. To assist doctors in diagnosis and treatment, an accurate segmentation of pelvic structure is essential. However, considering factors such as individual differences among different patients, bone mineral density unevenness, and narrowing of the hip joint space, it is difficult to rely only on gray scale information for accurate segmentation.

This paper proposes a novel segmentation method based on CT images of the pelvis. In the sequence of CT slices, because of the small shooting distance, the morphological characteristics between two adjacent CT images have high similarity. Thus, the key frames can be extracted. And the results of key frames are used to direct the segmentation of the remaining pelvic CT images according to the watershed algorithm based on skeleton markers. The proposed method greatly decreases the diagnostic time, and it’s helpful for the early diagnosis of patients, selection of surgical planning, etc.

## Methods

**Fig. 1:**
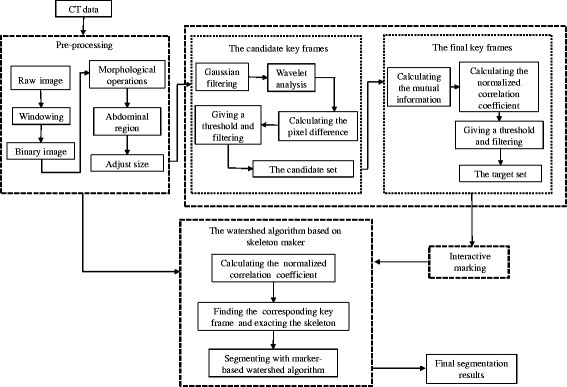
Schematic diagram of pelvic bone segmentation. The method consists of four parts: pre-processing of the CT image, key frame extraction, interactive marking and the watershed algorithm based on skeleton markers. After pre-processing, some CT images are selected as key frames. Experts manually add or remove marker points to realize the segmentation of key frames. The segmentation results of key frames will be used to segment other CT images

In this algorithm, we will select some CT images as key frames by using the method of key frame extraction. Then, experts will manually mark key frames to appoint the bone topological structure of these CT images. The segmentation results of key frames will be applied to describe the bone topology of other images. Next, the bone topology of the CT images will be used as a marker, and the watershed algorithm based on skeleton markers will be used to realize the automatic segmentation of pelvic structure. The algorithm consists of four parts: pre-processing of the CT image, key frame extraction in the CT image sequence, interactive marking and the watershed algorithm based on skeleton markers. The pretreatment process is used to denoise, extract regions of interest, and resize the image, reducing the amount of data in a single CT image. The CT sequence key frame extraction process is divided into two steps, and this process is used to reduce the number of CT images required for manual segmentation. In addition, the aim of interactive marking is to specify the bone topology of key frames. By matching key frames, the bone topology of each CT image can be acquired so that the accuracy of pelvic segmentation can be guaranteed. Finally, based on the interactive marking of key frames, the watershed algorithm based on skeleton markers is used to automatically segment all the CT images. The overall schematic diagram of the algorithm is shown in Fig. 1. In the following sections, the method is explained in detail.

### Dataset

The dataset has been obtained from Tianjin Medical University General Hospital and Tianjin Nankai Hospital. Data has been randomly collected from 20 patients with traumatic pelvic injuries. These patients are in the 35- to 60-year age range and both sexes account for half. For each CT image sequence, the size of the planning CT images in the axial plane is 512 × 512 pixels, with 1 mm image resolution and 2–7 mm slice thickness. Besides, a total of 245 images are collected from each patient.

### Pre-processing

Pre-processing removes surrounding artifacts from the original image, such as the CT platform and cables. Moreover, the image size is also adjusted in this process. The pelvis area is segmented from the original image as follows:

1. Only extract the image part of each DICOM file. The image data is 16 bits and there are 65,536 Gy levels. Considering the limited resolution of the human eyes and the limitations of experimental facilities, convert the data in the image using (1) with data compression to 256 Gy levels.

1 $$ f(x)=\left\{\begin{array}{c}0\\ {}\left[x-\left(c-\frac{w}{2}\right)\right]\times \frac{255}{w}\\ {}255\end{array}\begin{array}{c}x\le c-\frac{w}{2}\\ {}c-\frac{w}{2}<x<c+\frac{w}{2}\\ {}x\ge c+\frac{w}{2}\end{array}\right. $$

Here, *f* is a bitmap gray value, *x* is image data, *w* is window width, and *c* is window level.

2. Create a binary version of the original image according to (2).

2 $$ f(x)=\left\{\begin{array}{c}0\\ {}1\end{array}\right.{\displaystyle \begin{array}{c}x=0\\ {} else\end{array}} $$

3. Apply morphological operations to the binary image so that the different objects can be separated and that the object which has the largest area is the pelvis. Next, all the pixels in this object will be set to 1, and others become 0. The binary image is called the “mask image”.
4. Conduct an image convolution with the mask image and the original image, removing the CT platform, artifacts and other interference. At this time, the image is reserved for only the pelvis region; the remainder has been set to 0.
5. Adjust the size of the image by cutting out the non-pelvis area as much as possible. From now on, the pre-processed image is called the “input image”.

### The method of key frame extraction

**Fig. 2:**
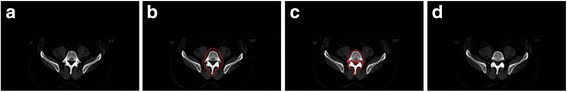
Four consecutive CT images of Patient 1. (**a**) and (**b**) have similar bone topologies. We believe that each has three bone topologies, while (**c**) and (**d**) have four obvious bone topologies. The differences are shown in red

After pre-processing, key frame extraction from the CT image sequence becomes the focus of the method. In this paper, key frames refer to the CT images of a CT sequence that have obvious changes in bone structure. As we can see from Fig. 2, there are four consecutive CT images. The first two CT images have similar bone structures. From the graph, we also find that the bone structure of (c) has obvious topological differences from the previous images. (b) has three bone topologies, while (c) has four obvious bone topologies. Therefore, (c) can be selected as a key frame. The method of key frame extraction consists of two steps. The first step is to obtain the candidate key frames. The last step is to obtain the final key frames.

To obtain candidate key frames, we extract the approximate region of bone as a region of interest so that the computation can be reduced. The pixel difference of the adjacent images is used to determine whether a new candidate key frame is required. The pixel difference of the adjacent images is defined as (3).

3 $$ Dif={y}_{j+1}-{y}_j $$

The greater the value is, the lower the degree of similarity is. Therefore, when the pixel difference of the adjacent images is greater than the threshold, it is considered that the difference between the adjacent images is large and that the similarity is not obvious. Thus, a new candidate key frame is added to the candidate set.

To summarize, the extraction of candidate key frames is implemented as follows:

**Table:** 

1. Use a Gaussian filter on the input image.	
2. Perform wavelet analysis of the filtered image and conduct image reconstruction using the approximation matrix. 3. Calculate *T*_1_ based on (4).	
4. Create a binary image based on (5).	
5. Convolute the binary image with the filtered image and the resulting image is called the “interesting image”.	
6. Sort the interesting images in accordance with the spatial order and import CT data{y_1_, y_2_, y_3_, ⋯, y_L_}.	
7. For *i* = 1 to *L* ‐ 1	
Calculate the pixel difference of the adjacent images	
If the difference is greater than *T*_2_	
the *i* + 1image is added to the candidate set	
Endif	
Endfor	
8. The candidate set of key frames {*g*_1_, *g*_2_, *g*_3_, ⋯, *g*_*l*_} is gained.	

During the extraction of the candidate key frames, *T*_1_ is the mean value of the reconstructed image of the non-zero area. It is defined as (4) and used to create a binary image. (4) and (5) are combined to obtain the approximate region of bones. In particular, the key frames in the candidate set are the pre-processed images and not the reconstructed images.

4 $$ {T}_1=\sum \limits_{x=0}^{N-1}\sum \limits_{y=0}^{M-1}f\left(x,y\right)/\left[N\times M- Zeros(S)\right] $$

Where *f*(*x*, *y*) is a bitmap gray value, *N* is the width of image, and *M* is the height of image. S = {(x, y)|*f*(*x*, *y*) = 0} is the set of background pixels and zero pixels. *Zeros(S)* is the notation used for the cardinality of set *S*.

5 $$ Mask\left(x,y\right)=\left\{\begin{array}{c}1\\ {}0\end{array}\right.{\displaystyle \begin{array}{c}f\left(x,y\right)>{T}_1\\ {}f\left(x,y\right)\le {T}_1\end{array}} $$

Where *Mask*(*x*, *y*)is the binary image of reconstructed image.

**Fig. 3:**
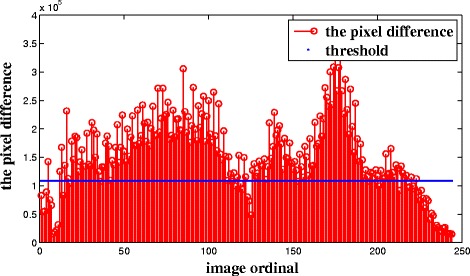
Pixel difference value of images for Patient 1

To illustrate the key frame extraction process, we will use data of Patient 1 to explain the process in detail. After generating the interesting images, these images are numbered from 1 to 245 according to the anatomic structure. Figure 3 shows the pixel difference value of the example. To avoid missing key frames, *T*_2_ is defined as (6), and it is described in blue in Fig. 3. After the extraction of the candidate set, there are 196 CT images to be selected out.

6 $$ {T}_2=0.7\times mean(Dif) $$

To obtain the target key frames, mutual information [31] and the normalized correlation coefficient [32] are utilized. The mutual information is defined as (7), and the normalized correlation coefficient is defined as (8). The mutual information reflects the gray correlation of the image; a greater mutual information value indicates that the gray difference of the two images is smaller. However, there is a lack of spatial location information so that redundant key frames will be extracted only when using mutual information. Thus, the normalized correlation coefficient is proposed to reduce the redundant key frames; a greater normalized correlation coefficient value indicates that the two images have higher similarity. The experimental results show that using the normalized correlation coefficient can effectively reduce the number of redundant key frames.

7 $$ I\left(x,y\right)=\sum \limits_{a\in {f}_1,b\in {f}_2}{P}_{f_1{f}_2}\left(a,b\right)\log \left[{P}_{f_1{f}_2}\left(a,b\right)/{P}_{f_1}(a){P}_{f_2}(b)\right] $$

Where ${P}_{f_1}(a)$ is the probability density of image*f*_1_, and ${P}_{f_2}(b)$ is the probability density of image *f*_2_. ${P}_{f_1{f}_2}\left(a,b\right)$ is the joint probability density of image *f*_1_ and image *f*_2_.

8 $$ R\left(x,y\right)=\frac{\sum \limits_{i=0}^{M-1}\sum \limits_{j=0}^{N-1}\left[{f}_1\left(i,j\right)-\overline{f_1}\right]\left[{f}_2\left(i,j\right)-\overline{f_2}\right]}{\sqrt{\sum \limits_{i=0}^{M-1}\sum \limits_{j=0}^{N-1}{\left[{f}_1\left(i,j\right)-\overline{f_1}\right]}^2\sum \limits_{i=0}^{M-1}\sum \limits_{j=0}^{N-1}{\left[{f}_2\left(i,j\right)-\overline{f_2}\right]}^2}} $$

Here, *f*_1_(*i*, *j*) is the pixel value of image *f*_1_, *f*_2_(*i*, *j*) is the pixel value of image*f*_2_, $\overline{f_1}$ is the average gray value of image *f*_1_, and $\overline{f_2}$ is the average gray value of image*f*_2_ .

The extraction of the target set from the candidate set is illustrated as follows:

**Table:** 

1. Import the candidate set{*g*_1_, *g*_2_, *g*_3_, ⋯, *g*_*l*_}.	
2. For *i* = 1 to *l* ‐ 1	
Calculate the mutual information	
If (*I*(*i* + 1, *i*) ≤ *T*_3_) the *i* + 1image is added to the intermediate set Endif Endfor 3. For *j* = 1to *k* ‐ 1	
Calculate the normalized correlation coefficient	
If (*R*(*j* + 1, *j*) ≤ *T*_4_)	
the *j* + 1image is added to the target set	
Endif	
Endfor	
4. The target set of key frames {*k*_1_, *k*_2_, *k*_3_, ⋯, *k*_*t*_} is gained.	

**Fig. 4:**
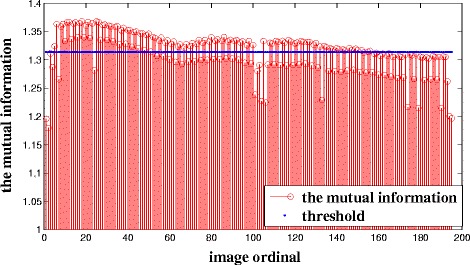
The mutual information value of candidate key frames for Patient 1

**Fig. 5:**
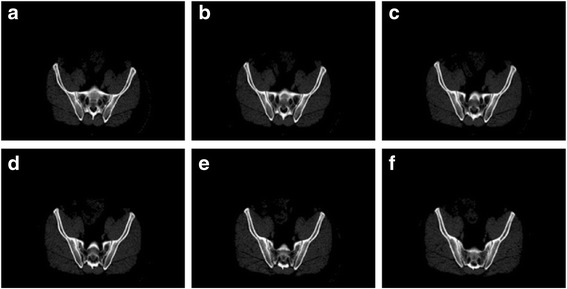
Example of some consecutive key frames in an intermediate set for Patient 1. After using the mutual information, some obtained key frames still have similar bone topologies. As seen from (**a** - **f**), all frames have three bone topologies

**Fig. 6:**
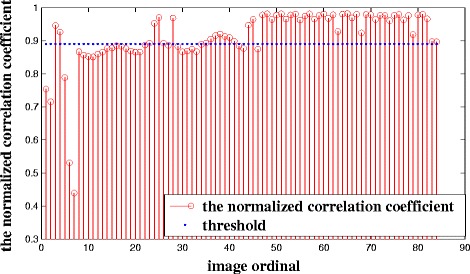
The normalized correlation coefficient of images in the intermediate set for Patient 1

The mutual information value of the candidate set is shown in Fig. 4. In this case, *T*_3_ is the mean mutual information value and is shown in blue. In addition, when the mutual information value of adjacent candidate key frames is less than*T*_3_, the candidate key frames will be selected out to calculate the normalized correlation coefficient. As we can see from Fig. 5, there is no significant change in the bone topology of these images in the intermediate set, and these images can use the segmentation results of the same key frame as the reference. Therefore, it is necessary to take measures to remove redundant key frames in the intermediate set. Figure 6 shows the normalized correlation coefficient value of images in the intermediate set. *T*_4_ is the mean normalized correlation coefficient and is shown in blue. An image will be chosen as a key frame in the target set when the normalized correlation coefficient is less than *T*_4_. Finally, there are 31 images in the target set.

**Fig. 7:**
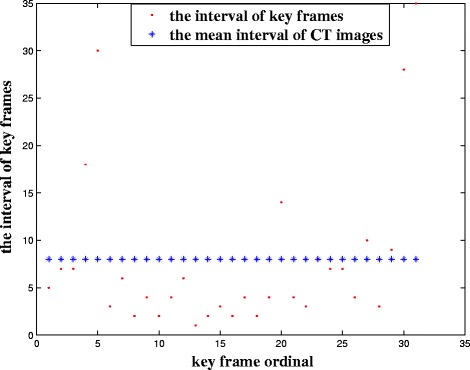
The interval between key frames in the target set for Patient 1

To describe the distribution of the key frames in the CT image sequence, Fig. 7 shows the interval between key frames. And the interval refers to the difference of each key-frame slice from the previous key-frame slice. At the same time, we equidistantly extract 31 images from the CT image sequence as a reference. It is found that the key frames are not evenly distributed. As a result, we can draw a conclusion that the key frames can effectively reflect the changes in pelvic structure and that it is meaningful to extract key frames.

Based on the above procedure, the target set of key frames {*k*_1_, *k*_2_, *k*_3_, ⋯, *k*_*t*_} can be extracted. After the extraction of key frames, the number of CT images required for manual marking is greatly reduced.

### Interactive marking

For each image in the target set, the first step is creating a binary version of the image. Next, marker points are obtained by extracting the skeleton from the binary image. The method of extracting the skeleton is introduced in the next section. Finally, the initial contours can be automatically generated through the watershed algorithm based on skeleton markers, which will also be introduced in next section. However, the results may be inconsistent with the topological structure of the human pelvis so that the segmentation results of this automatic algorithm cannot meet the requirements of clinical guidance. Therefore, the resulting image will be provided to experts, who will manually add or remove marker points to correct the initial contours. The segmentation results of key frames will be used to segment other CT images.

### The watershed algorithm based on skeleton marking

The method is divided into two parts: skeleton extraction and the marker-based watershed algorithm. Before segmentation, it is first necessary to search for the key frame matching the CT image. When a CT image is processed, the corresponding key frame image is obtained by calculating the normalized correlation coefficient between the CT image and key frames in the target set. In the corresponding key frame image, the bone region segmentation results will be regarded as foreground, and the remaining region will be regarded as background. Next, the skeleton of foreground and background can be obtained via skeleton extraction. On the other hand, the gradient image of CT image should be gained. Then, using skeleton image mark the gradient image and shield the original minimum pixels in gradient image. Therefore, the new gradient image will be acquired. Finally, the segmentation results are obtained by using watershed transform for the new gradient image.

#### Skeleton extraction

**Fig. 8:**
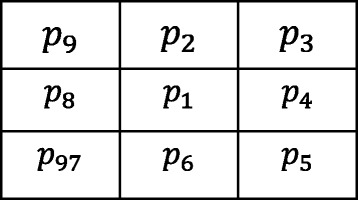
The relationship between neighboring pixels

The skeleton can provide information about the size and shape of targets in an image [33]. In this paper, we use the skeleton to describe location and topology information for pelvic bones. The method of thinning is used for skeleton extraction [34]. Whether or not a boundary point is converted into a background point is determined by the neighboring relations, and the iterative process is ended up with a pixel width. Supposing that the foreground pixel value is 1, the background pixel value is 0, *n*(*p*_1_) is the number of non-zeros in the neighborhood, and *s*(*p*_1_) is the total number of adjacent pixels from 0 to 1. Figure 8 shows the relationship between a pixel *p*_1_ and eight-neighboring pixels. Whether a point can be removed or not depends on the eight adjacent points. In this paper, we consider mainly the following three cases [35]: *υ*_1_ indicates whether the deletion will cause region splitting or not; *υ*_2_ indicates whether it is the east, south, or northwest border; *υ*_3_ indicates whether it is the north, west, or southeast border. These values can be described as in (9).

9 $$ {\displaystyle \begin{array}{l}{\upsilon}_1=\left\{{p}_1\left|2\le n\left({p}_1\right)\le 6,s\left({p}_1\right)\right.=1\right\}\\ {}{\upsilon}_2=\left\{{p}_1\left|{p}_2\times {p}_4\times {p}_6=0,{p}_8\times {p}_4\times {p}_6=0\right.\right\}\\ {}{\upsilon}_3=\left\{{p}_1\left|{p}_2\times {p}_4\times {p}_8=0,{p}_8\times {p}_2\times {p}_6=0\right.\right\}\end{array}} $$

10 $$ {\displaystyle \begin{array}{l}{D}_1=\left\{{p}_1\left|{p}_1=1,{p}_1\in {\upsilon}_1,{p}_1\in {\upsilon}_2\right.\right\}\\ {}{D}_2=\left\{{p}_1\left|{p}_1\in {\upsilon}_1,{p}_1\in {\upsilon}_3\right.\right\}\end{array}} $$

The skeleton extraction algorithm is divided into two steps. First, the boundary pixels that satisfy the condition *D*_1_ in the whole image are sought. The pixel points that we find are the ones that can be deleted. The pixels are converted to the background points after traversing the whole image. Then, the boundary pixels that meet condition *D*_2_ in the transformed image are sought. Similarly, the pixels are converted after traversing the whole image. After this process is the next iteration, until there are no points that can be deleted between the two steps. In addition, the two conditions are presented as in (10).

As for key frames, the skeleton of the foreground (*I*_*F*_) and background (*I*_*B*_) can be generated by utilizing the skeleton extraction algorithm. The skeleton is shown as *S*_*H*_ = *Skel*(*I*_*F*_, *I*_*B*_) and it will be the marker points for the marker-based watershed algorithm.

#### The marker-based watershed algorithm

The marker-based watershed algorithm [36] is based on a gradient image. Therefore, creating a gradient image is the first step in this process. In this method, the gradient image is obtained using the Sobel operator. The convolution templates (*S*_*x*_, *S*_*y*_) can be described as follows:

11 $$ {S}_x=\left[\begin{array}{ccc}1& 2& 1\\ {}0& 0& 0\\ {}-1& -2& -1\end{array}\right]\kern1.68em {S}_y=\left[\begin{array}{ccc}1& 0& -1\\ {}2& 0& -2\\ {}1& 0& -1\end{array}\right] $$

The gradient of pixel *p*_1_ can be gained by calculating the convolution of the templates and the eight neighboring pixels in Fig. 8. The solution is as follows:

12 $$ {\displaystyle \begin{array}{l}{G}_x={p}_9+2\times {p}_2+{p}_3-\left({p}_7+2\times {p}_6+{p}_5\right)\\ {}{G}_y={p}_9+2\times {p}_8+{p}_7-\left({p}_3+2\times {p}_4+{p}_5\right)\end{array}} $$

The gradient image can be described as *∇f*(*x*, *y*), and *∇f*(*x*, *y*) = [*G*_*X*_, *G*_*Y*_]^*T*^, where *G*_*x*_ is the gradient along the *X* direction, and *G*_*y*_ is the gradient along the *Y* direction.

The skeleton *S*_*H*_ is used to mark the gradient image *∇f*(*x*, *y*) At the same time, the original minimum pixels in gradient image will be shielded. In other words, in the marked gradient image, the local minimal value corresponds to the pixel region that is the non-zero area in the skeleton image. Finally, segmentation of the pelvic structure is achieved by using the watershed transform for the marked gradient image.

## Results

The proposed segmentation method is tested on 20 patients. Each patient has 245 pelvic CT images. Patient 1 is used as an example to display the results from different stages of the method and they are presented in this section as follows.

**Fig. 9:**
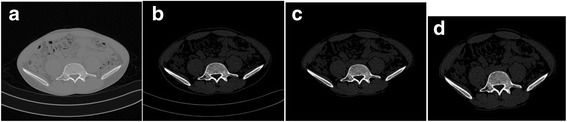
The pre-processing results of Patient 1. (**a**) is the original CT image whose pixel plane is 512 × 512. (**b**) is the image obtained by windowing, the window width is 600 Hu, and the window level is 900Hu. (**c**) is the pelvis area without the CT table, cables, etc. (**d**) is the pre-processed image. The image size is adjusted by cutting out the non-pelvis area as much as possible

Figure 9 shows the pre-processing results for the example. The results show that the edges of bone tissue are clearly defined by the window technology. Therefore, adjusting the window is necessary. In addition, the pelvis area can be extracted completely from the CT image after the removal of artifacts, platforms, etc.

**Fig. 10:**
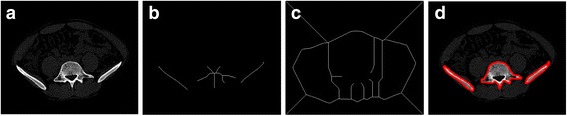
Example results of Patient 1 obtained by the watershed algorithm based on skeleton markers. (**a**) is the input image as well as the pre-processed image. (**b**) is the skeleton image the for foreground and indicates the location of the pelvic structure. (**c**) is the skeleton image for the background and indicates the location of the other structures. (**d**) is the segmentation result and it is shown in the pre-processed image. The automatically segmented pelvic structure contours are displayed in red

Figure 10 shows the results obtained by the watershed algorithm based on skeleton markers. The results show that the distribution of pelvic structure can be well described by the skeleton and that the pelvic structure can be segmented accurately in a single CT image using this method.

**Fig. 11:**
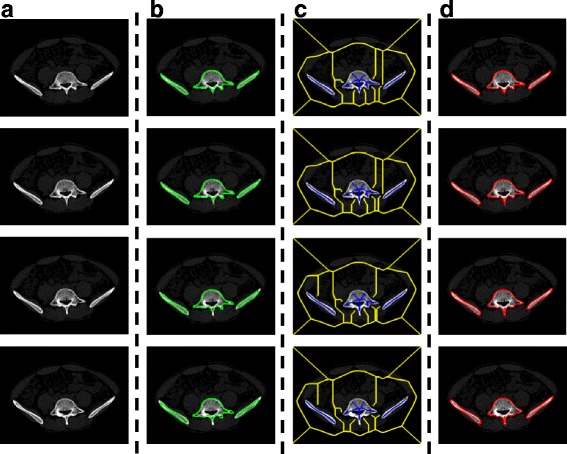
Example results of Patient 1 obtained by the proposed method. (**a**) is the input image as well as the pre-processed image. (**b**) is the manual segmentation results for the corresponding key frame, and the edges are shown in green. (**c**) is the skeleton images. The skeleton of foreground is shown in blue and the skeleton of background is displayed in yellow. These skeletons will be used as marker points for the marker-based watershed algorithm. (**d**) is the segmentation result of the proposed method and it is shown in the pre-processed image. The automatically segmented pelvic structure contours are displayed in red

Figure 11 shows the segmentation results of four CT images that are consecutive in the anatomic structure by the proposed method. The results show that the distribution of pelvic structure is well described by the marker points that were obtained from the corresponding key frame. Furthermore, the results also indicate that the proposed method can reliably segment the CT image sequence.

**Fig. 12:**
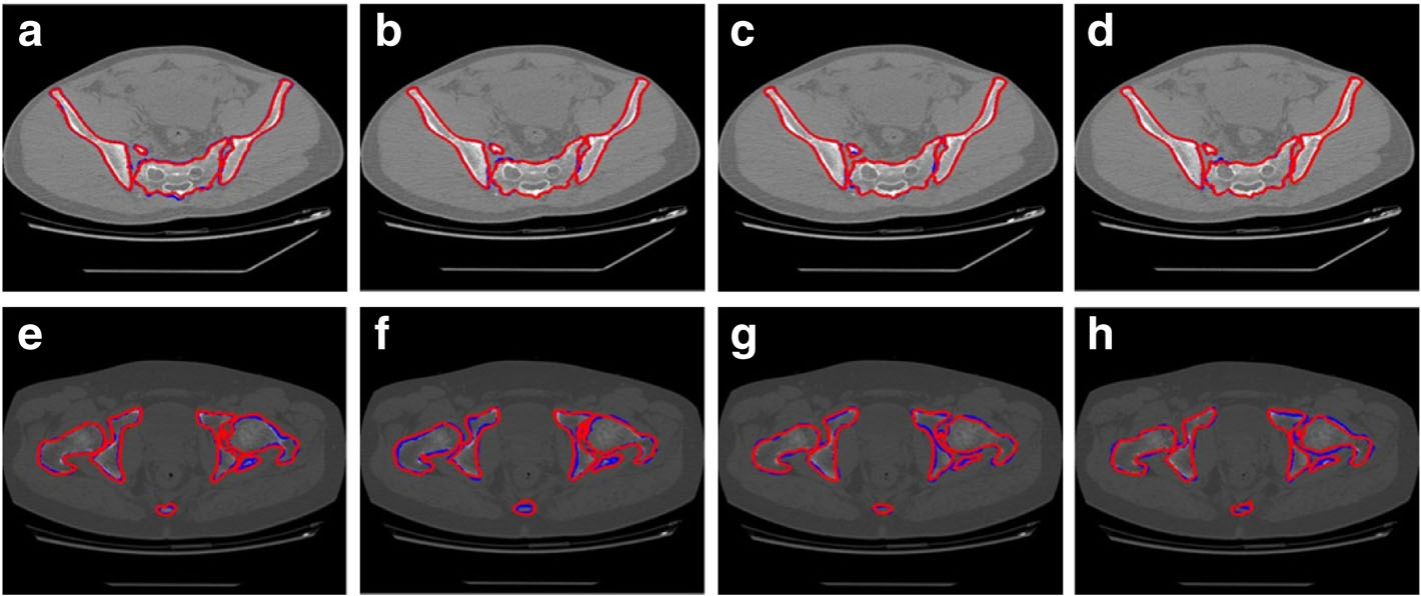
Example of segmentation results using proposed method in the fracture area. (**a**), (**b**), (**c**) and (**d**) are from Patient 3. (**e**), (**f**), (**g**) and (**h**) are from Patient 7. The segmentation results of the proposed method are shown in the original image. The manual segmentation results by experts are displayed in blue while the automatically segmented pelvic structure contours are displayed in red

Figure 12 shows the segmentation results using the proposed method in fracture areas. (a), (b), (c) and (d) are consecutive and these images are from Patient 3. These images show the segmentation results of CT images with sacrum bone fracture. (e), (f), (g) and (h) are also consecutive and they are from Patient 7. And they display the contours of bones in ischium bone fracture area. As can be seen from Fig. 12, the proposed method can accurately segment the fracture area.

Table 1 shows the number of key frames in each CT sequence. The results show that the ratio between the number of key frames and the total CT sequence number is approximately 13%. Thus, the number of images that need to be manually marked will be greatly reduced by extracting key frames.

**Table 1:** Shows the number of key frames in each CT sequence

Images	Total no. of CT images	Total no. of the candidate set	Total no. of the target set
Patient 1	245	178	28
Patient 2	245	153	32
Patient 3	245	148	30
Patient 4	245	164	33
Patient 5	245	157	33
Patient 6	245	177	31
Patient 7	245	169	32
Patient 8	245	163	27
Patient 9	245	157	31
Patient 10	245	182	35
Patient 11	245	178	31
Patient 12	245	159	27
Patient 13	245	170	30
Patient 14	245	170	33
Patient 15	245	144	29
Patient 16	245	138	27
Patient 17	245	169	31
Patient 18	245	173	33
Patient 19	245	165	29
Patient 20	245	154	30

## Discussion

In this section, some measures are taken to evaluate the performance of the proposed algorithm. A quantitative comparison is performed between the manual segmentation and the computed segmentation. In addition, a three-dimensional display of the segmented bone structure is rendered. Besides, the performance of the watershed algorithm based on skeleton markers and gradient vector flow (GVF) is compared. And the running time of the proposed algorithm is also an important index.

### Evaluation measures

The overlapping area and the mean deviation distance are used to quantify the accuracy [37]. In this section, 245 CT images will be manually segmented by experts for each patient and the results will be the ground truth.

*S*_1_ represents surface obtained by the proposed method and *S*_2_ represents surfaces obtained by experts manually. Besides, *L*_1_ represents a set of contour points obtained by the proposed method and*L*_2_ represents a set of contour points manually obtained by experts. As for two surfaces, we define *A*_1_ as the area for surface *S*_1_and *A*_2_as the area for surface *S*_2_.

The overlapping area *O* of two surfaces *S*_1_ and *S*_2_ is defined as:

13 $$ O=\frac{A_1\cap {A}_2}{A_2}\times 100\% $$

The mean deviation distance *Mad* of two contour points sets *L*_1_ and *L*_2_ is defined as:

14 $$ Mad=\frac{1}{K}\sum \limits_{n=1}^Kd\left({l}_n,{L}_2\right) $$

Where *l*_*n*_ denotes each contour point in*L*_1_,*K*denotes the total number of contour points in *L*_1_. Deviation distance refers to the distance between the contours obtained by the proposed algorithm and the ground truth. The deviation distance between *L*_1_ and *L*_2_is defined as:

15 $$ d\left({l}_n,{L}_2\right)={\min}_{l_i\in {L}_2}{\left\Vert {l}_n-{l}_i\right\Vert}_2 $$

Five patients of the 20 patients are randomly selected, and their CT images are used to calculate the overlapping area *O*. The shapes presenting with an overlapping area of more than 90% are classified as “accurate”, the shapes presenting with an overlapping area of 90–80% are classified as “fair”, and the shapes presenting with an overlapping area of less than 80% are classified as “unacceptable” [37].

Table 2 shows the segmentation results and the overlapping area of CT images from five patients’. The results show that the average overlapping area of all the data is more than 94%. More than 93.5% of the results are classified as “accurate”, and less than 2% of the results are classified as “unacceptable”. The unacceptable results may be caused by blurred edges of bones, uneven gray value of bones, lack of appropriate key frames, etc.

**Table 2:** Shows the segmentation results and the overlapping area of CT images from five patients’

Images	The average overlapping area	Accurate	Fair	Unacceptable
Patient 1	96.3%	95.1%(233/245)	3.7%(9/245)	1.2%(3/245)
Patient 2	94.1%	93.5%(229/245)	4.5%(11/245)	2%(5/245)
Patient 3	97.4%	96.7%(237/245)	2.9%(7/245)	0.4%(1/245)
Patient 4	94.8%	94.7%(232/245)	4.1%(10/245)	1.2%(3/245)
Patient 5	95.6%	95.9%(235/245)	3.3%(8/245)	0.8%(2/245)

**Fig. 13:**
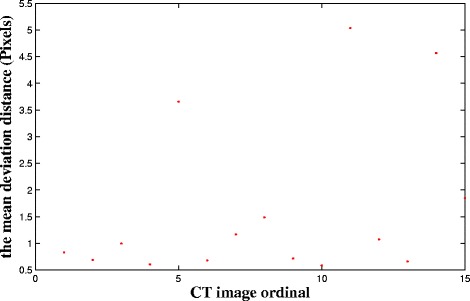
The mean deviation distance of fifteen testing images

Figure 13 shows the mean deviation distance of fifteen testing images. These images are randomly selected from Patient 1 to Patient 5.The maximum mean deviation distance is 5.03 pixels, and the minimum mean deviation distance is approximately 0.58 pixels. Without the impact of the method, an unsmooth contour may also lead to a large mean deviation distance. The results show that the proposed method is reliable for accurate pelvic segmentation.

**Fig. 14:**
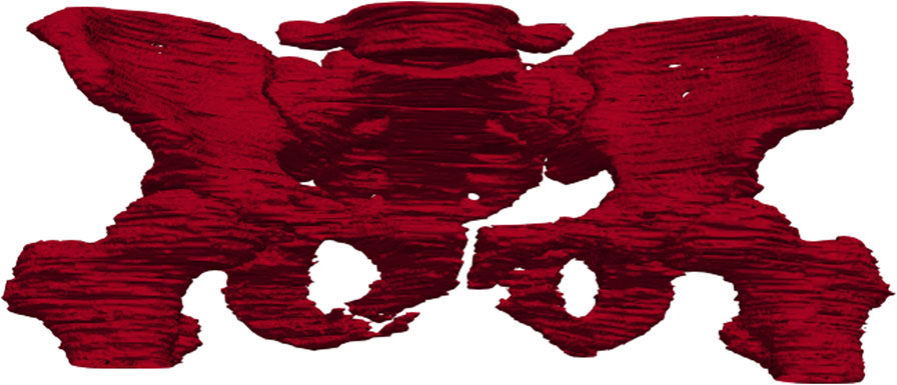
Three-dimensional pelvic bone structure of Patient 4

After segmented sequences of CT slice images are obtained, a three-dimensional model of pelvic bone structure can be reconstructed utilizing these segmented bone structures. In addition, three-dimensional visualization may be used for further validating the accuracy of the proposed method. The segmentation results are visually inspected as shown in Fig. 14. As can be seen from this figure, the three-dimensional structure of pelvic bone is clearly presented and the detected fracture exits in the pelvic region.

### The gradient vector flow (GVF) model

In this section, the segmentation performance using the watershed algorithm based on skeleton markers and the GVF model is compared. The GVF model was introduced by Xu and Prince [38] in 1998. In relation to the traditional snake model, the sensitivity of the initial curve position and the convergence of the concave area are improved. In this paper, the segmentation results of the key frames are taken as the initial curve in the GVF model.

**Fig. 15:**
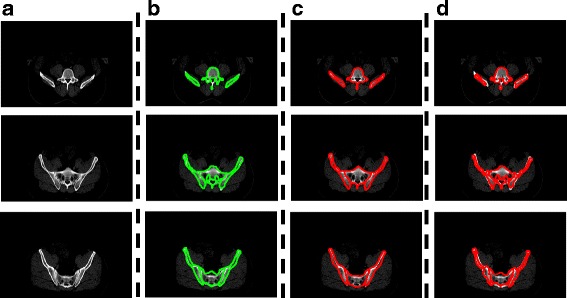
Example results using the watershed algorithm based on skeleton markers and the GVF model. These testing images are from Patient 1. (**a**) is the input image as well as the pre-processed image. (**b**) is the manual segmentation results of the corresponding key frame, and the edges are shown in green. (**c**) is the automatic segmentation results of the watershed algorithm based on skeleton markers, and the edges are shown in red. (**d**) is the segmentation results of the GVF model, the edges are shown in red and they are shown in the pre-processed image

Figure 15 shows the example of segmentation results using the two methods. As can be seen from Fig. 15b, the results of key frames cannot accurately describe the edges of pelvic bones in intervening images. However, the contours of bones in key frames can offer location and topology information of pelvic bones in the intervening images. Location information can be used to indicate the possible areas of bones and the topology information can be used to indicate the possible number of bones. From Fig. 15d, we can find that the information given by key frames cannot make GVF model obtain good segmentation results. Through this comparison of the segmentation results, it can be found that the watershed algorithm based on skeleton markers has better segmentation effect than the GVF model.

### Algorithm running time

In this section, the test environment is as follows: the CPU is Inter-Core i3–2120 3.3 GHZ, the system memory is 4 GB, and the programming environment is MatLabR2014. Table 3 shows the running time of the proposed method except for the time of manual marking. As we can see from the graph, the average running time is approximately 2 min for one patient with 245 CT images.

**Table 3:** Shows the Running Time of the Proposed Method

Procedure	Average Running Time
Pre-processing	27.657s
Extraction of candidate key frames	36.288s
Extraction of final key frames	45.482s
The watershed algorithm based on skeleton markers	21.834s
Total algorithm	131.261s

Interactive marking has an approximate time of 30 s for each key frame image because the experts only need to manually add or remove a small number of marker points. The experimental results show that there are approximately 30 CT images that are selected for manual marking. Therefore, the proposed method takes approximately 15 min for one patient with 245 CT images. Such performance suggests a considerable reduction in processing time compared with manual segmentation.

In conclusion, the proposed method not only ensures the accuracy of pelvic segmentation but also reduces the time of segmentation.

## Conclusions

A novel segmentation method based on CT image sequences of the pelvis is presented in this paper. The key parts of the method are the method of key frame extraction and the watershed algorithm based on skeleton markers. By using the method of key frame extraction, the number of manually marked CT images decreases to approximately 30. Based on the method of skeleton extraction, the distribution of pelvic structure can be well described in key frames. Considering that anatomical structures have characteristics of spatial continuity, the skeleton can be used to describe the location and topological information of pelvic bones in other CT images. Appling the watershed algorithm based on skeleton markers can achieve accurate, fast and efficient segmentation of pelvic structure. And segmentation results not only provide an important reference for early diagnosis and decisions regarding surgical procedures, they also offer more accurate data for medical image registration, recognition and 3D reconstruction.

In this paper, the performance of the proposed method depends on the accuracy of key frame extraction. When a crucial CT image is not selected, the accuracy of segmentation using the method will be affected. In addition, the performance is also affected by blurred edges of bones, variation of bones, etc. since the watershed algorithm is applied.

Future work will focus on the following points: 1) Propose a more efficient method to extract key frames. This method should ensure that the key frames are not redundant and that crucial CT images will not be missing. In addition, the running time of the method should also be considered. 2) Apply this method to segment other organs. 3) Using this method to process MRI and ultrasound images. 4) Explore a method for 3D reconstruction relying only on the key frames.

In conclusion, the proposed method is able to achieve accurate, fast, and efficient segmentation of pelvic CT image sequences.

## Abbreviations

CT: Computed tomography
GVF: The gradient vector flow (GVF) model
